# Optimizing Swine Oral Fluid Sampling Procedures for Growing Pigs in Commercial Settings

**DOI:** 10.3390/pathogens13121097

**Published:** 2024-12-12

**Authors:** Grzegorz Tarasiuk, Marta D. Remmenga, Kathleen C. O’Hara, Marian K. Talbert, Sarah Mielke, Marisa L. Rotolo, Pam Zaabel, Danyang Zhang, Jeffrey J. Zimmerman

**Affiliations:** 1 Department of Veterinary Diagnostic and Production Animal Medicine, College of Veterinary Medicine, Iowa State University, Ames, IA 50011, USA; tarasiuk@iastate.edu; 2 USDA: VS: Strategy and Policy, Center for Epidemiology and Animal Health, Fort Collins, CO 80526, USA; 3 National Pork Board, Des Moines, IA 50325, USA; mrotolo@pork.org (M.L.R.);; 4 Department of Statistics, College of Liberal Arts and Sciences, Iowa State University, Ames, IA 50010, USA; zhangdy@iastate.edu

**Keywords:** oral fluids, behavior, pen size, surveillance, sampling, swine

## Abstract

Pen-based oral fluids are used extensively for surveillance and disease detection in swine, but there is sparse information on the sampling process itself. To address this shortcoming, we documented the pen-based oral fluid sampling process with the aim of optimizing the number of pigs in a pen that contributed to the sample. We quantified the effects of (1) previous experience with rope sampling (training), (2) the number of ropes suspended in the pen, and (3) sampling time on pig participation and pig-rope contact. A subset of pigs was clearly marked for individual identification and their interactions with ropes video recorded. Thereafter, pig-rope contacts were counted from the recordings, with “contact” defined as an individually identified pig clearly taking the rope into its mouth. Data were analyzed using appropriate models (R version 4.4.1 R core team 2024). Training, provision of additional ropes, and extended sampling time all increased pig participation across pen sizes. However, for routine oral fluid collection in the field, we recommend training pigs prior to hanging ropes for sample collection and increasing sampling time to maximize the pigs’ contribution to the oral fluid sample. Importantly, these studies focused on pig behavior and not detection; thus, future studies should evaluate the impact of these same factors on the probability of detection.

## 1. Introduction

The swine industry is continually working to improve all aspects of pork production; the development of efficient, yet diagnostically sensitive, disease detection and surveillance methods is a significant part of this ongoing effort. First described in 2008 for the surveillance of porcine reproductive and respiratory syndrome virus (PRRSV) and porcine circovirus type 2 (PCV2) [1,2], pen-based oral fluids are widely used because of the reduced labor and cost inputs required compared to individual pig samples [3]. That is, pen-based oral fluid samples are easily collected with limited stress to workers or pigs and, when collected from a population, are more likely to detect the pathogen of interest than individual pig samples [3].

As reviewed by Munguía-Ramírez et al. (2023) [4], individual animal and pen-based oral fluids are amenable to the detection of a wide variety of viral and bacterial pathogens of swine, including some of the most economically impactful, e.g., African swine fever virus, classical swine fever virus, foot-and-mouth disease virus, PCV2, PRRSV, pseudorabies virus, swine influenza virus, and others.

Under experimental conditions, studies have shown that pathogen-specific nucleic acids are detectable in individual animal oral fluids beginning shortly after exposure. For example, Kittawornratt et al. (2010) [5] reported that 10% (7/69) of PRRSV-inoculated boars produced RNA-positive individual animal oral fluids one day post-inoculation (DPI), 76% (52/68) of boars on DPI 2, 94% (66/70) of boars on DPI 3, and 100% (67/67) on DPI 4. Likewise, individual animal oral fluids contain both systemic and locally produced antibodies. Thus, PRRSV antibodies were consistently detected in individual animal oral fluids as soon as 7 DPI [6].

Further, oral fluids include diagnostic targets from the environment that pigs acquire through normal feeding and/or exploratory behaviors and subsequently deposit in the oral fluid sample. This was first described by Johnson et al. (2012) [7] who showed that the ELISA-detectable PRRSV antibodies in oral fluid samples from a PRRSV-negative production system were actually derived from the porcine-derived dry-sprayed plasma included in the pigs’ feed. Subsequently, Tarasiuk et al. (2024) [8] documented the process by placing 20 mL of a fluorescence tracer in the middle of a pens of 30, 60, and 125 pigs and detecting it in pen-based oral fluid samples.

For these reasons, oral fluids are widely used by producers and veterinary practitioners. For example, in 2023, 6 Midwest US diagnostic laboratories reported that 133,821 of the case submissions they received included oral fluid samples to be tested by polymerase chain reaction (PCR) for one or more of 8 pathogens (influenza A virus, *Mycoplasma hyopneumoniae*, PCV2, PCV3, porcine delta coronavirus, porcine epidemic diarrhea virus, PRRSV, and transmissible gastroenteritis virus) [9].

Despite its widespread use, we have an incomplete understanding of pig behavior in the context of pen-based oral fluid sampling. This information is fundamental to optimizing oral fluid sampling in commercial swine production systems and enhancing its role in surveillance. Therefore, the aim of this research was to explore pig behavior with the goal of optimizing the number of pigs in a pen that contributed to an oral fluid sample. To that end, we successively quantified the effect of previous experience with rope sampling (“training”), varying the number of ropes suspended in the pen, and increasing sampling time on pig participation in oral fluid sampling. Notably, this research focused on pig behavior and not on diagnostic target detection; albeit higher pig participation in oral fluid collection would likely lead to a higher probability of population-based detection.

## 2. Materials and Methods

Three studies were performed in pens of 10- to 14-week-old cross-bred pigs (Chester White, Large White, Duroc, Landrace) in commercial wean-to-finish farms located in the Midwest US. Barns were tunnel ventilated, fully slatted, and housed 1200 to 5000 animals each. The interior of the barns was divided into pens using gates that allowed nose-to-nose contact among pigs in neighboring pens. Pens were equipped with standard feeders and nipple drinkers and held between 25 and 130 pigs, as specified for each study.

Treatments were performed at the pen level. Treatments were randomly assigned to pens within barns using online software (random.org accessed on 17 April 2022); with the exception that hospital and recovery pens were excluded from selection. Oral fluid sampling was performed using 3-strand twisted 100% cotton rope 1.3 cm (0.5 inches) (Skydog Rigging Equipment, Lake in the Hills, IL, USA) placed 150 cm (59.1 inches) from the corner(s) of the pen to be sampled, with the end of the rope suspended at the pigs’ shoulder height. With the exception of Study 1, all studies were done in pens of pigs with prior experience with rope sampling (“training”). Immediately prior to administering treatments, a convenience sample of pigs in each pen was clearly marked with colored livestock dye (Ideal^®^ Prima^®^ Spray-On, Neogen Corp., Lansing, MI, USA) to provide unambiguous visual identification of individual animals.

Pig sampling behavior was quantified and analyzed in these studies in terms of pig-rope contacts and participation. “Contact” was defined as one individually identified pig clearly taking the rope into its mouth and “participation” was the percentage of individually identified pigs in a pen that interacted with the rope. “Mean contact time” was calculated from participant pigs over the observation period, i.e., non-participant pigs were excluded.

Contact data was based on video recordings taken during the observation period using video cameras (GoPro^®^ 7 cameras, GoPro Inc., San Mateo, CA, USA) strategically placed at each rope location. To generate contact data, video recordings were viewed, and contacts counted for each individually identified pig. Contact counts were based on one-minute intervals, i.e., one or more contacts with a rope in the same minute was counted as “one”. Thus, one pig observed for 30 min had a maximum of 30 possible contacts. It follows that “contact time” for an individual pig was defined as the number of contacts over the observation period.

### 2.1. Study 1 (Training Effect)

The objective of Study 1 was to quantify the effect of pigs’ previous experience with rope sampling (“training”) on subsequent interactions with ropes. The effect of training on mean contact time and pig participation was assessed in 16 pens, each holding approximately 125 pigs, by comparing pre- vs. post-training data. To perform the study, 15 pigs were individually marked with colored livestock dye, a rope was suspended on the walkway side of the pen, and pig contacts were video recorded for 60 min. This procedure was the repeated the following day, after pigs had been “trained” via prior rope exposure. Because the colored dye was impermanent, a new convenience sample of 15 pigs was marked in each pen for the post-training sampling.

### 2.2. Study 2 (Number of Ropes per Pen by Pen Size)

The objective of Study 2 was to quantify the effect of the number of pigs in the pen (pen size) and number of ropes available to the pigs in the pen on mean contact time and pig participation observed over a 30-min sampling period. Circulation of pigs among ropes when multiple ropes were available was also evaluated for Study 2 and was based on the percent of marked pigs that contacted more than one rope when 2 or more ropes were available.

The study was performed in 128 pens holding approximately 25, 65, 100, or 130 pigs (32 pens for each pen size). Three pigs were individually marked for observation in pens of 25, 9 pigs were marked in pens of 65, 12 pigs were marked in pens of 100, and 15 pigs were marked in pens of 130 pigs. Four treatments (1, 2, 3, or 4 ropes in the pen) were evaluated and each treatment was randomly applied to 8 different pens in each pen size category. Ropes were placed uniformly in all pens, i.e., first on the walkway side of the pen (ropes 1 and 2) and then on the opposite side of the pen (ropes 3 and 4).

### 2.3. Study 3 (Sampling Time by Pen Size)

The objective of Study 3 was to quantify the effect of prolonged sampling on participation by analyzing mean contact time and pig participation over a 90-min observation period using one rope per pen in pens of 30, 90, and 120 pigs (32 pens per size category).

The study was conducted in one barn of 32 pens, each pen holding 120 pigs. Initially, each of the 32 large pens was randomly assigned to a size category (120 pigs in one large pen or two pens of 30 and 90 pigs). One rope was hung in each pen and, for every pen size, 20 individually identified pigs were tracked over the 90-min observation period. Seven days later, the study was repeated but pen sizes were reversed relative to the first sampling, i.e., the intact pens holding 120 pigs were divided and those that had been divided into 30 and 90 pigs were left intact.

### 2.4. Data Analysis

The primary aims of the analyses were to describe mean contact time and pig participation as a function of training (Study 1), pen size and number of ropes provided (Study 2), and pen size and sampling time (Study 3). Analyses were based on the observed behavior (contacts) of individually identified pigs (observational unit) in pens (experimental unit). Data were analyzed in R (version 4.4.0. R core team 2024) [10]. Appropriate models were fit to predict pen values for each factor combination of interest and reported with a 95% prediction interval using the base R package, lme4() and merTools() [11,12].

Because the proportion of pigs that interacted with the rope was bounded by 0 and 1, a generalized linear model with a binomial link was fit to the pig participation data with pen as a random factor for all three studies using lme4() [11]; prediction intervals (PI) were generated by bootstrap methods using merTools() [12]. Analysis of pig participation data for Studies 1 and 2 did not detect significant interactions between factors; therefore, models were fitted without interactions. Significance was defined as *p*-value < 0.05.

Mean (pen level) contact time data were analyzed using a generalized linear model with assumptions of normality [10]. Analysis of contact time for Study 1 and Study 3 included pen size*sampling time interaction while the analysis for Study 2 did not because no significant interaction was detected. In Study 2, the percentage of marked pigs that contacted more than one rope when 2 or more ropes were available (“circulation”) was analyzed using a generalized linear model with a binomial link. Interactions were not significant for circulation and thus a model without interactions was fit.

In Study 3, contact behavior over time was described by calculating the percent of marked pigs that contacted the rope in specific time periods (minutes 1–30, 31–60, and/or 61–90). As shown in Table 1, this produced 7 distinct contact behavior categories (A–G). These labels are used later to present results of the data analysis. The data were analyzed using a generalized linear model with assumptions of normality. The analysis showed a significant pen size*contact behavior category interaction, and this was included in the model.

## 3. Results

Study 1 quantified the effect of training (previous experience with ropes) on contact time and participation in 16 pens (125 pigs each) with observations made on 15 marked pigs in each pen. Compared to untrained pigs, training significantly increased participation (*p* < 0.001, Table 2), i.e., each 10 min of sampling increased participation by 3.5% in untrained pigs vs. 4.3% in trained pigs. Mean contact time was significantly lower among trained (mean = 5.9 min) vs. untrained (mean = 8.5 min) participant pigs (*p* < 0.001) at 60 min. It should be noted that Studies 2 and 3 were conducted in trained pigs.

Study 2 quantified participation, contact time, and “circulation” among ropes, i.e., pigs’ use of more than one rope when ≥2 ropes were available, as a function of the number of pigs in a pen (pen size) and the number of ropes provided. The study was performed in 128 pens holding 25, 65, 100, or 130 pigs (32 pens for each pen size) and was based on a 30-min sampling time. The number of individually marked pigs varied by pen size (approx. 12% per pen): 3 pigs in pens of 25, 9 pigs in pens of 65, 12 pigs in pens of 100, and 15 pigs in pens of 130 pigs.

Participation was affected both by the number of ropes (*p* < 0.001) and pen size (*p* < 0.001), but no significant rope*pen size interaction was detected (Table 3). Participation decreased by 4% for each additional 10 pigs per pen and increased by 6.8% for each additional rope available in the pen.

**Figure 1:**
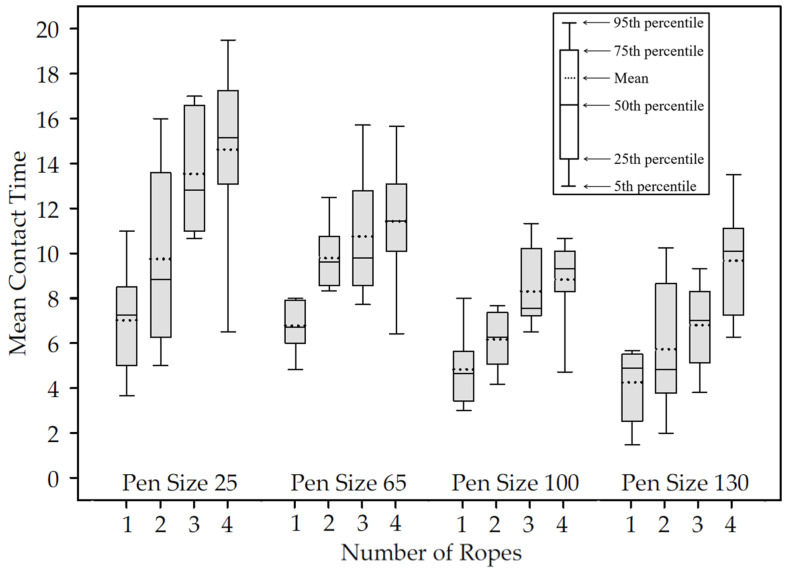
Study 2. Observed pen-level mean contact time of marked pigs by pen size and number of ropes provided for oral fluid collection for a 30-min sampling time. (Data based on 3 marked pigs in pens of 25; 9 in pens of 65; 12 in pens of 100; 15 in pens of 130).

As shown in Figure 1, contact time was significantly affected by the number of ropes provided (*p* < 0.001) and pen size (*p* < 0.001) with a significant interaction (*p* = 0.039). For example, in pens of 25 pigs, mean contact time was 7.7 min when one rope was provided vs. 14.8 min with four ropes. By comparison, mean contact time with one rope was 4.4 min vs. 8.4 min with four ropes in pens of 130 pigs.

“Circulation”, i.e., contact with more than one rope when 2 or more ropes were available, was dependent on the number of ropes available in the pen (*p* < 0.001) and pen size (*p* < 0.001). Overall, the smaller the pen size, the more circulation among ropes was observed. As the number of ropes increased, the percentage of pigs that interacted with more than one rope also increased, but this percentage declined as pen size increased.

Thus, when 2 ropes were available, 38%, 19%, 17%, and 9% of marked pigs interacted with more than one rope in pens of 25, 65, 100, and 130, respectively. When 3 ropes were available, 58%, 47%, 22%, and 11% of marked pigs used more than one rope in pens of 25, 65, 100, and 130, respectively and, with 4 ropes, the estimates were 79%, 47%, 48%, and 28%.

Study 3 (Table 4) quantified participation in pens holding 30, 90, and 120 pigs (32 pens per size category) over a 90-min observation period, with 20 individually identified pigs observed in each pen. Both sampling time and pen size affected participation (*p* < 0.001) and contact time (*p* < 0.001). Specifically, participation increased over time in all pen size categories but decreased by 2.8% for each additional 10 pigs.

Mean contact time for pen sizes of 30, 90, and 120 pigs were 9.0, 7.2, and 6.3 min after 30 min of sampling, 14.7, 10.4, and 8.3 min after 60 min, and 20.3, 13.7, and 10.4 min after 90 min, respectively.

Participant pigs’ contact behavior over a 90-min observation period is presented in Table 5 in terms of the 7 contact behavior categories defined in Table 1. For all pen sizes (30, 90, 120 pigs per pen), the largest proportion of pigs were in category A, i.e., contacted the rope ≥ 1 times in each time period (minutes 1–30, 31–60, and/or 61–90). Specifically, Category A included 51%, 38%, and 31% of the pigs for pen sizes of 30, 90, and 120 pigs, respectively. Within each pen size, the proportion of pigs in Category A was significantly larger than other categories (*p* <0.001). Importantly, the proportion of pigs in Category G (pigs with their first rope contact in the last 30 min of sampling) was 12%, 19% and 23% for pen sizes of 30, 90, and 120 pigs, respectively.

## 4. Discussion

Although oral fluid specimens are widely used in surveillance, there are few publications that describe the pig behaviors associated with the collection process. Among these, White et al., (2014) [13] reported that >70% of pigs participated in oral fluid collection (one rope, 30 min sampling time) in pens holding approximately 25 pigs. Similarly, Graage et al. (2019) [14] stated that 76.5 to 81.9% of pigs participated in the oral fluid collection in pens holding 16 to 30 pigs. Likewise, Seddon et al., (2012) [15] reported that over 80% of pigs participated in oral fluid collection in pens holding 17 to 24 pigs during a 60-min sampling time. Thus, estimates of pig participation in oral fluid collection in pens holding 17 to 30 pigs have been consistent across studies, but among U.S. producers, Tarasiuk et al. (2024) [16] found that the average pen size for grow-to-finish farms was 75 to 82 pigs.

Producers and veterinarians need information on pig behaviors associated with oral fluid collection over a range of pen sizes to inform standardized oral fluid sampling protocols appropriate to their production facilities and the surveillance objectives. To meet that need, we conducted 3 independent studies to quantify the effect of (1) previous experience with rope sampling (training), (2) the number of ropes suspended in the pen, and (3) sampling time on pig-rope interactions.

Study 1 showed that training in pens holding 125 pigs significantly increased pig participation, i.e., 56% of trained pigs participated in oral fluid sampling vs. 35% of untrained pigs in a 60-min sampling (Table 2). Results of Study 2 also showed that the mean contact time was less in the trained pens than the untrained pens, perhaps allowing for the increased pig participation. These results were consistent with a report by White et al. (2014) [13] in which 75.5% of trained pigs versus 54.4% of untrained pigs participated in oral fluid over a 30-min sampling period in pens of 25 pigs. Thus, training has a strong positive effect on participation.

Study 2 (Table 3) showed that both the number of ropes and pigs per pen significantly affected pig participation and contact time. That is, pig participation increased as the number of ropes increased, for example, in pens of 25 pigs, 70% of marked pigs participated in oral fluid sampling when one rope was provided vs. 89% when four ropes were provided. In a pen of 130 pigs, 25% of marked pigs participated in oral fluid sampling when one rope was provided vs. 53% when four ropes were provided. Mean contact time increased as the number of ropes increased. For example, in pens of 25 pigs, mean contact time was 7.7 min when one rope was provided vs. 14.8 min with four ropes; mean contact time with one rope was 4.4 min vs. 8.4 min with four ropes in pens of 130 pigs.

Study 3 showed that longer sampling time (90 min) with one rope would significantly increase pig participation in oral fluid sampling. For example, in pens of 30 pigs, 55% of marked pigs participated in oral fluid sampling over a 30-min sampling period vs. 73% of marked pigs over 90-min. In pens of 120 pigs 30% of marked pigs participated in oral fluid sampling over a 30-min sampling period vs. 48% of marked pigs over 90-min. Mean contact time, regardless of pen size, increased as sampling time increased. For example, in pens of 30 pigs the mean contact time was 9.0 and 20.3 min after 30 and 90 min of sampling, and in pens of 120 was 6.3 and 10.4 min after 30 and 90 min of sampling.

In particular, Studies 2 and 3 showed that pig participation in pen-based oral fluid sampling can be increased with more ropes per pen and with longer sampling time. Study 2 showed that more circulation was observed when more ropes were available, particularly in smaller pens. In other words, in small pens, the more ropes available, the more frequently a given pig would chew on multiple ropes. In contrast, in larger pens, while some circulation was observed, providing multiple ropes captured “new” pigs. This finding emphasizes the potential value of hanging multiple ropes to increase pig participation, particularly in large pens.

In the field, the time available to sample, the pathogen, the surveillance objective, and the cost of testing additional oral fluid samples will drive the choices for maximizing pig participation in pen-based oral fluid sampling. In addition, Study 3 showed that, regardless of pen size, some pigs, especially in the largest pen size, did not contact the rope until the final 30 min of the 90-min observation period. Specifically, in pens of 30, 90, and 120 pigs, 12%, 19%, and 23%, respectively, of the marked pigs did not participate in oral fluid sampling until minutes 61 to 90. Longer sampling time increased pig participation by providing the limited resource (rope) for longer time, allowing more pigs the opportunity to contact it. Therefore, longer sampling time is one practical approach to maximizing pig participation in oral fluid sampling.

In the way of a general comment, it should be noted that the effect of pen size on participation in Studies 2 and 3 can be partially understood in terms of competition for a limited resource. A pig’s interaction with a rope hung in its pen is limited by physical access. That is, depending on the size of the pigs and the manner in which the rope is presented, there is only enough space for 4 to 6 pigs to touch the rope at any given time. Logically, as the number of pigs in a pen increases, the number of “competitors” likewise increases. Therefore, some pigs that are interested in the rope are unable to compete successfully for access.

Competition for rope access does not mean that larger pens are unsuitable for oral fluid sampling. Extrapolating the results of Study 3 to a barn of growing pigs (Table 6), assuming a 30-min sampling, 6 oral fluid samples from pens holding 30, 90, or 120 pigs each would contain “contributions” from approximately 97, 213, and 237 pigs, respectively. Under the same circumstances, increasing the sampling time to 60 min would increase participation to approximately 111, 257, and 292 pigs and increasing the sampling time to 90 min would increase participation to approximately 124, 298, and 346 pigs, respectively. Pragmatically, these numbers are far beyond the individual pig samples that could be collected on a routine basis for an ongoing surveillance program.

## 5. Conclusions

Swine oral fluids are usually collected for one of two reasons: (1) to monitor a target (pathogen or live vaccine) in a population or (2) to demonstrate the negative status of a population, e.g., gilts or boars in isolation. Previous publications recommended collecting oral fluids by providing one rope per pen for ~30 min for trained and 45 to 60 min for untrained pigs [3]. In alignment with previous studies, we recommend one rope per pen and training the pigs prior to the first sampling, but our new data supports sampling for ≥60 min to maximize pig participation. Of course, there are many reports of successful detection based on 20- to 30-min sampling, including the original reports on PRRSV and PCV2 surveillance [1,2]. Longer sampling allows more pigs to deliver diagnostic targets to the oral fluid sample [8] when one rope was provided, which hypothetically should provide a higher probability of detection. Alternatively, hanging more ropes, particularly in the larger pen sizes, also allows more pigs to deliver diagnostic targets to the oral fluid sample. In the field, practical constraints will drive the approach used to maximize pig participation in pen-based oral fluid sampling.

## Figures and Tables

**Table 1:** Categories used in the analysis of Study 3 contact behavior data.

Intervals	Contact Category Criteria						
	A	B	C	D	E	F	G
1–30 min	Contact	Contact	Contact	Contact	No	No	No
31–60 min	Contact	Contact	No	No	Contact	Contact	No
61–90 min	Contact	No	No	Contact	No	Contact	Contact

**Table 2:** Study 1—Effect of training on pig participation ^1^ in oral fluid sampling across a 60-min observation period.

Treatments ^2^	No. Ropes	Cumulative Participation (Predicted Percent, 95% PI)		
		At 20 min	At 40 min	At 60 min
Untrained	1	21% (3, 66)	27% (5, 73)	35% (7, 80)
Trained	1	39% (8, 83)	48% (12, 87)	56% (15, 91)

^1^ Study conducted in 16 pens of 125 pigs each. Participation defined as percentage of individually identified pigs (15 pigs per pen) that interacted with the rope. ^2^ Training was associated with increased participation (*p* < 0.001).

**Table 3:** Study 2—Predicted pig participation in oral fluid sampling by pen size and number of ropes in the pen over a 30-min observation period.

Pen Size ^1^	No. Ropes	Cumulative Participation (Predicted Percent, 95% PI) ^2^		
		At 10 min	At 20 min	At 30 min
25 pigs	1	53% (13, 90)	63% (17, 93)	70% (21, 95)
	2	64% (19, 93)	72% (25, 96)	78% (31, 97)
	3	73% (15, 91)	80% (33, 97)	84% (38, 98)
	4	80% (35, 97)	86% (42, 98)	89% (48, 99)
65 pigs	1	36% (7, 80)	46% (9, 87)	53% (12, 90)
	2	47% (6, 79)	56% (12, 90)	63% (14, 92)
	3	57% (15, 91)	66% (21, 94)	72% (25, 96)
	4	67% (21, 94)	75% (27, 96)	79% (30, 97)
100 pigs	1	24% (4, 71)	32% (6, 80)	37% (7, 83)
	2	32% (6, 79)	42% (9, 85)	47% (10, 88)
	3	42% (9, 85)	52% (12, 89)	57% (14, 91)
	4	53% (14, 90)	63% (19, 94)	66% (23, 95)
130 pigs	1	16% (2, 58)	22% (4, 69)	25% (4, 72)
	2	22% (4, 68)	30% (5, 77)	33% (6, 80)
	3	31% (5, 76)	40% (8, 83)	43% (8, 84)
	4	40% (8, 83)	50% (11, 88)	53% (12, 89)

^1^ Each pen size category (25, 65, 100, or 130 pigs) consisted of 32 pens. Three pigs were individually marked for observation in pens of 25, 9 pigs were marked in pens of 65, 12 pigs were marked in pens of 100, and 15 pigs were marked in pens of 130 pigs; ^2^ Pen size (*p* < 0.001) and number of ropes (*p* < 0.001) affected participation at 10, 20, and 30 min of sampling; no interaction between pen size and number of ropes was detected (*p* > 0.15).

**Table 4:** Study 3—Predicted pig participation in oral fluid sampling by pen size over a 90-min observation period at defined time points.

Pen Size ^1,2^	No. Ropes	Cumulative Participation (Predicted Percent, 95% PI) ^2^		
		At 30 min	At 60 min	At 90 min
30 pigs	1	55% (15, 89)	64% (20, 93)	73% (27, 95)
90 pigs	1	37% (8, 81)	47% (11, 86)	57% (16, 90)
120 pigs	1	30% (6, 75)	38% (8, 81)	48% (11, 86)

^1^ Each pen size category (30, 90, or 120 pigs) consisted of 32 pens. 20 individually marked pigs were observed in each pen for 90 min. ^2^ Pen size (*p* < 0.001) and time (*p* < 0.001) affected participation at 30, 60, and 90 min of sampling; no interaction (pen size*time) was detected (*p* > 0.66).

**Table 5:** Study 3—Predicted participant pig contact behavior over a 90-min observation period.

Pen Size ^1^	Percent (95% PI) of Pigs That Contacted the Rope by Contact Category ^2,3^						
	A	B	C	D	E	F	G
30 pigs	51% (31, 72)	11% (0, 32)	12% (0, 33)	15% (0, 35)	7% (0, 28)	17% (0, 37)	12% (0, 32)
90 pigs	38% (18, 58)	16% (0, 36)	15% (0, 35)	15% (0, 35)	11% (0, 32)	21% (1, 42)	19% (0, 40)
120 pigs	31% (11, 52)	18% (0, 39)	16% (0, 37)	15% (0, 36)	13% (0, 34)	24% (3, 44)	23% (3, 44)

^1^ Estimates for each pen size category (30, 90, or 120 pigs) based on observations in 32 pens. Specifically, 20 individually marked pigs were observed in each pen for 90 min. ^2^ Participant pigs were assigned to one of seven categories, as defined in Table 1. ^3^ The model predicted the category values without restricting their sum to 100%. Lower bounds were restricted to zero which may result in prediction intervals with less than 95 percent coverage.

**Table 6:** Extrapolation to a barn housing growing pigs: number of pigs predicted to participate in oral fluid sampling by pen size, number of pens sampled, and sampling time.

Pen Size	No. of Pens Sampled	Predicted Number of Participant Pigs (95% PI) ^1^		
		30 min	60 min	90 min
30 pigs	4	65 (36, 92)	74 (44, 99)	83 (55, 105)
	6	97 (60, 133)	111 (73, 143)	124 (89, 153)
90 pigs	4	143 (67, 229)	170 (88, 255)	199 (111, 281)
	6	213 (111, 326)	257 (148, 366)	298 (185, 406)
120 pigs	4	159 (65, 272)	193 (91, 310)	231 (120, 343)
	6	237 (117, 381)	292 (159, 444)	346 (199, 493)

^1^ Bootstrap results were simulated based on the generalized linear model with a binomial link used to analyze the data given in Table 4.

## Data Availability

The data that support the findings of this study are available on request from the corresponding author.
